# Mechanical Thrombectomy for Tandem Occlusions of the Internal Carotid Artery—Results of a Conservative Approach for the Extracranial Lesion

**DOI:** 10.3389/fneur.2018.00928

**Published:** 2018-11-05

**Authors:** Alexandre Blassiau, Matthias Gawlitza, Pierre-François Manceau, Serge Bakchine, Isabelle Serre, Sébastien Soize, Laurent Pierot

**Affiliations:** ^1^Department of Neuroradiology, Hôpital Maison-Blanche, Reims, France; ^2^Department of Neurology, Hôpital Maison-Blanche, Reims, France

**Keywords:** tandem lesions, ischemic stroke, mechanical thrombectomy, carotid artery stenting, large vessel occlusion, conservatory approach, percutaneous transluminal angioplasty (PTA)

## Abstract

**Background:** Mechanical thrombectomy (MT) is of clinical benefit for patients with extracranial-intracranial tandem lesions of anterior circulation. However, the optimal approach to the cervical lesion of the internal carotid artery (ICA) during MT has yet to be established. Data on a conservative approach for the proximal lesion during the acute phase are scarce.

**Methods:** A retrospective study on an institutional, prospective database was conducted. We included patients with anterior circulation stroke presenting with a tandem lesion that was approached conservatively during MT.

**Results:** Thirty-five 35 patients were included, of whom 25 (71.4%) had an atheromatous ICA lesion and 10 (28.6%) a dissection. Despite implementing a conservative strategy, acute percutaneous transluminal angioplasty (PTA) and/or stenting was necessary in 8 (22.9%) and 3 patients (8.6%), respectively. Of 27 surviving patients, 7 (25.9%) underwent delayed treatment of the ICA lesion. No new embolic events occurred between MT and delayed treatment. A favorable clinical outcome (mRS ≤ 2) was achieved in 15/35 patients (45.7%) and was associated with higher baseline ASPECTS (OR 1.62, 95% CI 1.08–2.45, *p* = 0.002) and successful recanalization (OR 9.39, 95% CI 1.92–45.80, *p* = 0.0005). Successful recanalization (TICI ≥ 2B) itself was observed in 54.3% of patients and was more likely with acute treatment of the proximal ICA lesion (OR 6.3, 95% CI 11–35.67, *p* = 0.03) and, more importantly, by the use of distal access catheters (OR 16.25, 95% CI 3.06–86.41, *p* = 0.0001).

**Conclusion:** A conservative approach for ICA lesions during MT is feasible and offers favorable outcomes and successful recanalization for a significant proportion of patients. However, acute treatment of the cervical lesion is often necessary (31.4%) to make the distal occlusion accessible. Clinical outcome is influenced by the size of the baseline ischemic core and by successful recanalization; the latter is strongly favored by the use of distal access catheters to pass the proximal lesion. The fact that acute treatment of the ICA lesion favored intracranial recanalization but had no effect on clinical outcome is probably due to sample size, emphasizing the need for large scale, randomized studies to determine the optimal treatment strategy for this pathology.

## Introduction

Tandem extracranial-intracranial lesions of the anterior circulation, i.e., the simultaneous occurrence of an intracranial large vessel occlusion and a high-grade stenosis or occlusion of the ipsilateral proximal internal carotid artery (ICA), account for 10–20% of large vessel strokes (1, 2). This pathology is particularly challenging as response rates to intravenous thrombolysis are low and the prognosis is often poor (1, 3).

Although patients with tandem lesions have been excluded from the majority of randomized studies in recent years, the HERMES analysis confirmed the beneficial effect of mechanical thrombectomy (MT) for this pathology (4). Thus, evidence indicates that patients with tandem lesions should not be excluded from MT.

Nonetheless, it has yet to be established how the proximal ICA lesion should be approached during MT. There are several possibilities, i.e., stenting, percutaneous transluminal angioplasty (PTA) alone or no treatment of the lesion; yet, all approaches present advantages and drawbacks. Stenting in the acute phase offers a definitive treatment in one step, but necessitates antiplatelet therapy–often in conjunction with intravenous thrombolysis–putting the patient at potentially elevated risk of intracranial hemorrhage or reperfusion injury (5–8). While simple PTA of the carotid lesion obviates acute antiplatelet therapy, a risk of reperfusion injury persists and the rate of significant restenosis is high (9). However, delayed treatment of the lesion by endarterectomy or stenting can be utilized in patients with favorable clinical outcomes, which may also help to reduce the number of unnecessary procedures (10). Not treating the cervical lesion at all has similar potential advantages and in addition may be faster, while eliminating the risk of reperfusion injury. Nevertheless, this strategy must be balanced against the drawback of a potential risk of recurrent cerebral embolism (11).

Recent meta-analyses did not find significant differences in clinical outcomes between patients treated with stenting and patients treated with PTA alone in the acute phase (12, 13). Wilson et al. concluded that most studies “have entirely or partly treated extracranial occlusion with stenting during initial treatment” and that very few focus on angioplasty-only approaches (12). To the best of our knowledge, data on a completely conservative approach for the proximal ICA lesion are even scarcer (14), which is why we aimed to augment the existing literature with our retrospective case series.

## Materials and methods

### Study design and ethics

The present paper is a retrospective study on our institutional, prospectively assembled thrombectomy database between October 2010 and October 2017. Inclusion criteria were patients with anterior circulation stroke presenting with a tandem extracranial and intracranial lesion (defined as a carotid stenosis of ≥90% or occlusion of the proximal carotid artery with simultaneous occlusion of an intracranial vessel of the anterior circulation), pretreatment imaging (MRI or CT) and clinical follow—up with 90 day modified Rankin Scale (mRS) assessment. Demographic data, vascular risk factors, admission National Institutes of Health Stroke Scale scores (NIHSS), stroke side, symptom onset to groin puncture and onset to reperfusion, MRI/CT and angiographic variables, information about delayed treatment of the carotid lesion by stenting or endarterectomy and the mRS score at 3 months were collected. Favorable outcome was defined as mRS 0–2. In line with French law, retrospective studies do not require ethics committee approval.

### Imaging evaluation

Brain MR examinations were performed on a 3-T MR unit (Skyra, Siemens, Erlangen, Germany; Achieva 2.1, Philips Healthcare, Best, The Netherlands) and included at least DWI, FLAIR, T2^*^ and TOF-MRA. CT scans were obtained on a tomograph with 64 slices using helicoid acquisition (General Electrics, Chicago, IL, United States).

Two interventional neuroradiologists, each with 8 years of experience, analyzed preinterventional cross-sectional imaging. Preinterventional CT- and DWI-ASPECT scores were evaluated and discrepancies resolved by consensus between readers. Additionally, all postinterventional neuroimaging studies performed during the patients' hospital stays were evaluated for the presence of intracranial hemorrhage (parenchymal hematoma PH1 and PH2) according to the European Cooperative Acute Stroke Study 3 (ECASS3) (15).

The cervical lesion was categorized on DSA as either atheromatous stenosis or dissection, according to typical angiographic imaging characteristics.

For the purpose of the study, two readers (8 and 20 years of experience), blinded from clinical and MRI data, assessed the intracranial occlusion site and initial and final thrombolysis in cerebral infarction (TICI) score on anonymized angiographic records. Successful recanalization was defined as TICI 2B-3 (more than two-thirds of the middle cerebral artery (MCA) territory) (16).

### Mechanical thrombectomy

Patients with acute ischemic stroke, presenting within 6 h of symptom onset with occlusion of a large intracranial vessel (ICA and/or MCA) and without initial intracranial hemorrhage were eligible for MT. Thrombectomy was performed after neuroradiologists and vascular neurologists made a multidisciplinary decision; in exceptional cases, the time window was extended beyond 6 h post symptom onset. Procedures were performed under conscious sedation using 6 F or 8 F catheters introduced via the common femoral artery. An initial angiographic run with the catheter placed in the common carotid artery confirmed the presence of a cervical ICA lesion. Our institutional protocol has evolved over time in line with technical advances. At the outset of the inclusion period, we passed the tandem lesion either with the guide catheter or, if the former was not possible, with the microcatheter, and performed an intracranial thrombectomy using a stent-retriever (Solitaire, FR, Medtronic, Minneapolis, MN, United States) in conjunction with syringe-aided aspiration on the guide catheter. The advent of flexible distal access and aspiration catheters changed this practice and treatment was modified by attempting to pass the stenosis with the distal access catheter, occasionally preceded by an aspiration maneuver at the level of the cervical lesion; intracranial stent-retriever thrombectomy was sometimes preceded or followed by a direct aspiration maneuver with 5 MAX, ACE 64, or ACE 68 reperfusion catheters aided by a mechanical pump providing continuous negative pressure (Penumbra, Alameda, CA, United States).

Our institutional protocol favors minimal treatment for the proximal ICA lesion and prefers balloon angioplasty using over-the-wire (Gateway, Boston Scientific, Fremont, CA, United States) or rapid-exchange (Sterling, Boston Scientific, Fremont, CA, United States) non-compliant PTA balloons only for patients in whom intracranial access through the proximal carotid lesion is not possible. Carotid stenting in the setting of a thrombectomy is considered only for patients (a) in whom intracranial access is not possible even after balloon angioplasty, and (b) in whom hemodynamic compromise due to insufficient collateral flow through the anterior and posterior communicating arteries is to be expected, which is typically investigated on preinterventional cross-sectional imaging studies and, in case of doubt, on a vertebral artery and a contralateral ICA angiogram after intracranial thrombectomy. Emergent carotid stenting can also be considered by the treating physician in case of a floating intraluminal thrombus in the ICA.

Delayed treatment of the cervical lesion by endarterectomy or stenting is typically considered in case of a favorable clinical outcome and with regard to the patient's comorbidities, life expectancy, compliance and personal preferences.

### Anticoagulation/antiaggregation

In case of an entirely conservative treatment and in case of balloon angioplasty of an atheromatous cervical lesion, antiaggregation with 75 mg of acetylsalicylic acid per day is started at discretion of the stroke neurologist and is maintained lifelong in the setting of a secondary prophylaxis.

Medical treatment of non-stented carotid artery dissection is conducted complying with the publication of Engelter et al. (17) and the duration of antiplatelet or anticoagulation therapy is guided by MRI control images.

In case of extracranial carotid stenting, the institutional protocol consists of intravenous administration of 250 mg of acetylsalicylic acid before deploying the stent. The day after the procedure, a cranial computed tomography and a craniocervical computed tomography angiography are conducted and in the absence of stent occlusion, intracranial hemorrhage and complete hemispheric infarction a loading dose of 300 mg of clopidogrel is administered per os. Oral dual antiplatelet therapy with 75 mg of both acetylsalicylic acid and clopidogrel is maintained during 3 months and acetylsalicylic acid monotherapy is continued thereafter.

### Statistical analysis

Continuous variables are described as mean ± SD or median with interquartile range (IQR) and categorical variables as number and percentage. Contingency analyses for categorical variables were performed using the exact Chi-square test. Continuous study parameters were compared among patients by either Student's *t*-test in case of a normal distribution or by the Mann-Whitney *U*-test in case of a non-normal distribution. To identify markers of favorable clinical outcome and successful revascularization, group comparisons and univariate logistic regression analyses were carried out. Due to the rather small sample size with resulting large confidence intervals, multivariate logistic regression models were not applied. All analyses were conducted using the JASP 9.0 freeware for Mac OS X (http://www.jasp-stats.com) and MedCalc for Windows (MedCalc Software, Ostend, Belgium).

## Results

### Study population

During the study period, 269 patients underwent MT in our department. Of these, 36 patients (13.4%) presented with an extracranial/intracranial tandem occlusion. One patient with a tandem lesion did not undergo MT due to an anesthesiology complication and was removed from the study population leaving 35 patients for statistical analysis. Demographic aspects of the study population are detailed in Table 1. Twenty patients (57.1%) presented with a left hemispheric stroke. Median baseline ASPECTS was 6. MT was performed in conjunction with IVT in 23 patients (65.7%) with a mean onset-to-groin time of 255.5 ± 53 min. The mean NIHSS was 17.3 ± 4.5. Intracranial occlusion sites were the carotid-T in 15 patients (42.9%), the MCA M1 segment in 19 patients (54.3%) and the MCA M2 segment in 1 patient (2.8%), respectively.

**Table 1:** Demographic characteristics of the study population.

**Male sex n/N (%)**	**30/35 (85.7%)**
Age (mean ± SD; range)	64.6 ± 11.2; 40–86 years
Left hemispheric stroke n/N (%)	20/35 (57.1%)
Baseline NIHSS (mean ± SD; range)	17.3 ± 4.5; 7–25
Baseline ASPECTS (median (IQR); range)	6 (4); 1–9
Onset to groin time (mean ± SD; range)	255.5 ± 53.0; 150–380 min
Onset to recanalization (mean ± SD; range)	336.7 ± 68.6; 230–440 min
Intravenous thrombolysis n/N (%)	23/35 (65.7%)
Hypertension n/N (%)	21/35 (60%)
Tobacco n/N (%)	10/35 (28.6%)
Diabetes mellitus n/N (%)	7/35 (20%)
Dyslipidemia n/N (%)	12/35 (34.3%)
Atrial fibrillation at admission n/N (%)	5/35 (14.3%)

### Clinical and angiographic outcomes

At 3 months, mRS scores were 0 in 3 (8.6%), 1 in 7 (20%), 2 in 6 (17.1%), 3 in 5 (14.3%), 4 in 5 (14.3%), and 5 in 1 (2.9%) patients. Eight patients did not survive, resulting in a mortality rate of 22.9%. A favorable outcome (mRS 0–2) was achieved in 16 of 35 patients (45.7%).

TICI 3 was achieved in 5 (14.3%), TICI 2B in 14 (40%), TICI 2A in 10 (28.6%) and TICI 0-1 in 6 patients (17.1%). The rate of successful intracranial recanalization was thus 54.3%. Distal access catheters were employed in 18 patients (51.4%). Emboli to previously unaffected territories occurred in 5 patients (14.3%) of which 2 were observed in patients treated without (2 of 17; 11.8%) and 3 in patients treated with a distal access catheter (3 of 18; 16.7%). The rate of postinterventional parenchymal hematoma was 5.7% (2/35) for PH1 and 5.7% (2/35) for PH2.

### Management of tandem lesions

Twenty-five patients (71.4%) were diagnosed with an atheromatous lesion and 10 patients (28.6%) with a dissection of the cervical ICA.

In 24 patients no acute treatment of the cervical lesion was performed (68.6%), whereas PTA only or stenting with or without concomitant PTA was carried out in 8 (22.9%) and 3 patients (8.6%), respectively. No statistically significant difference was observed for rates of acute treatment of the carotid lesions in atheromatous (9 of 25; 36%) or dissecting (2 of 10; 20%) lesions (*p* = 0.357).

**Figure 1:**
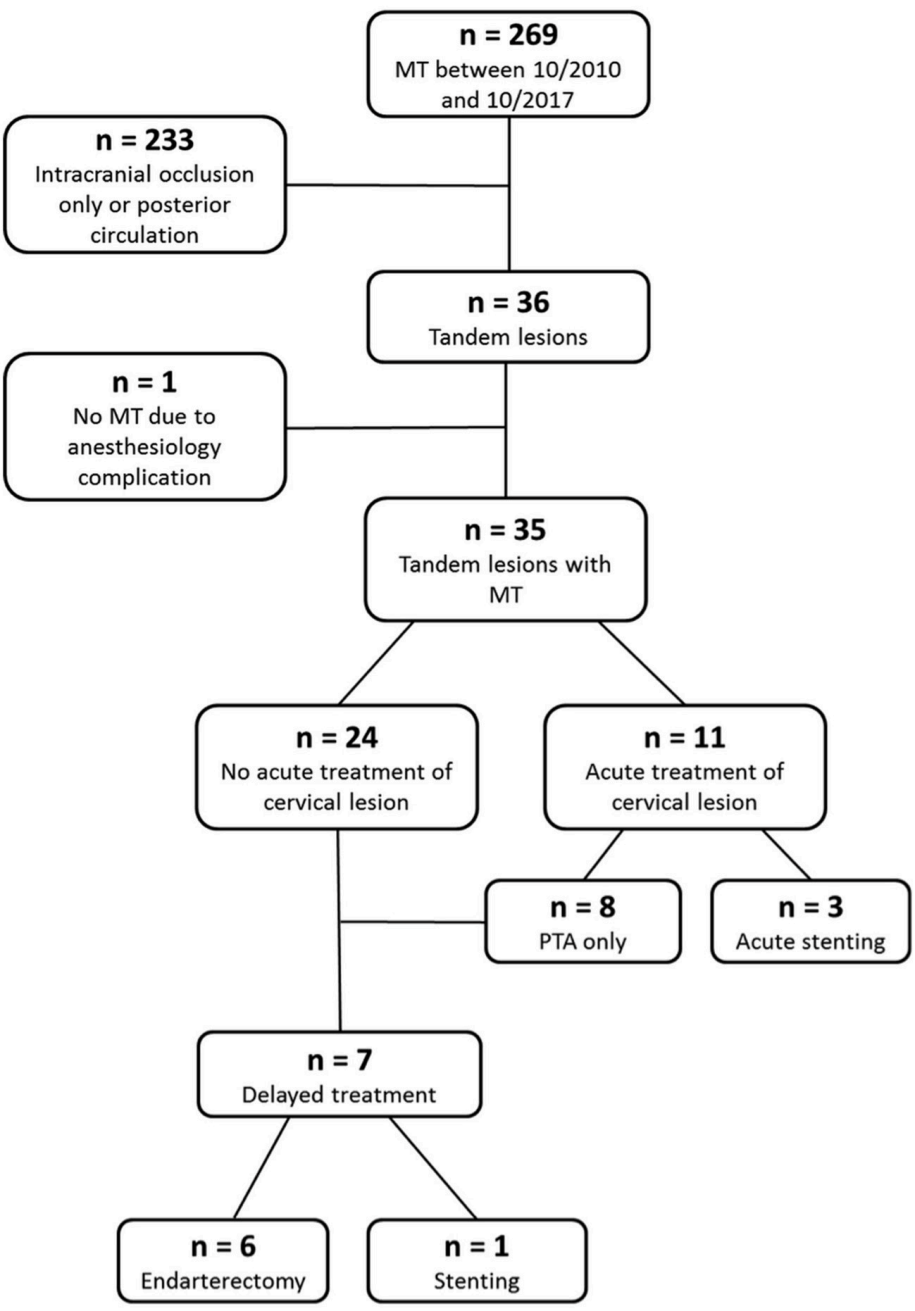
Flowchart of the study population.

Of 27 surviving patients, 7 (25.9%) underwent delayed treatment of the cervical lesion after a mean period of 41.7 ± 38.4 days (range 5–118). Delayed treatment consisted of carotid endarterectomy in 6 of 27 (22.2%) and carotid stenting in 1 of 27 patients (3.7%) and was only performed for atheromatous lesions of the ICA (7 of 20; 35%). The flowchart of the study population is depicted in Figure 1.

No neurological deterioration between the acute phase and the time of the delayed treatment was observed, and no neurological complications occurred during or after delayed treatment of the carotid stenosis.

### Factors influencing outcome and recanalization

The comparison of patients with favorable and unfavorable outcome at 3 months of follow-up is shown in Table 2. Notably, patients with favorable outcome had higher median ASPECTS on preinterventional imaging (7.5 vs. 5; *p* = 0.013) and experienced successful recanalization more often (81.3 vs. 31.6%; *p* = 0.006). All other cofactors were not statistically different between groups; specifically, no differences with respect to the etiology of the carotid lesion, the baseline NIHSS and the symptom onset to groin time were observed. Similar results were obtained by univariate logistic regression analysis (Table 3): The only significant factors associated with a favorable patient outcome were higher baseline ASPECTS values (OR 1.62, 95% CI 1.08–2.45, *p* = 0.02) and successful recanalization (OR 9.39, 95% 1.92–45.80, *p* = 0.0005). Moreover, a trend toward improved patient outcomes was observed when IV thrombolysis was employed (OR 3.09, 95% CI 0.89–18.28, *p* = 0.08).

**Table 2:** Favorable vs. unfavorable outcome.

	**Unfavorable outcome**	**Favorable outcome**	***P*-value**
**PATIENT CHARACTERISTICS**			
Male sex n (%)	16/19 (84.2%)	14/16 (87.5%)	1.0
Age (mean ± SD)	66.7 ± 12.9	62.1 ± 8.4	0.23
Left hemispheric stroke n/N (%)	9/19 (47.4%)	11/16 (68.8%)	0.31
Baseline NIHSS (mean ± SD)	17.7 ± 3.7	16.8 ± 5.3	0.55
Baseline ASPECTS (median (IQR), range)	5 (4, 1–9)	7.5 (2.25, 3–9)	0.013
Onset-to-groin time (mean ± SD)	264.3 ± 53.3	245.0 ± 53.1	0.29
Onset to recanalization (mean ± SD)	345.2 ± 69.8	326.7 ± 68.0	0.44
Intravenous thrombolysis n (%)	10/19 (52.6%)	13/16 (81.3%)	0.15
**ANGIOGRAPHIC VARIABLES**			
TICI ≥ 2B n (%)	6/19 (31.6%)	13/16 (81.3%)	0.006
Stenosis acutely treated n/N (%)	5/19 (26.3%)	6/16 (37.5%)	0.72
Distal access catheter n/N (%)	8/19 (42.1%)	10/16 (62.5%)	0.31
Atheromatous lesion n/N (%)	14/19 (73.7%)	11/16 (68.8%)	1.0
**OCCLUSION SITE**			
Carotid-T n/N (%)	10/19 (52.6%)	5/16 (31.3%)	0.31
**POSTPROCEDURAL CHARACTERISTICS**			
PH2 n/N (%)	2/19 (10.5%)	0/16 (0%)	0.49
Delayed treatment of ICA n/N (%)^*^	1/11 (9.1%)	6/16 (37.5%)	0.18

Significant differences are indicated with bold font.

* *in patients with mRS < 6*.

**Table 3:** Univariate logistic regression analyses with odds ratios for favorable outcome and successful recanalization.

	**Favorable outcome OR** **(95% CI)**	***P*-value**	**Successful recanalization** **OR (95% CI)**	***P*-value**
**PATIENT CHARACTERISTICS**				
Male sex	1.31 (0.19–9.02)	0.78	1.96 (0.28–13.50)	0.49
Age	0.96 (0.90–1.02)	0.22	0.98 (0.92–1.04)	0.52
Left hemispheric stroke	2.44 (0.61–9.80)	0.20	
Baseline NIHSS	0.95 (0.81–1.11)	0.53	0.95 (0.82–1.11)	0.59
Baseline ASPECTS	1.62 (1.08–2.45)	0.02	
Onset-to-groin time	1.01 (0.99–1.02)	0.28	0.99 (0.98–1.01)	0.37
Onset to recanalization	1.00 (0.99–1.01)	0.42	
Intravenous thrombolysis	3.09 (0.89–18.28)	0.08	0.77 (0.19–3.18)	0.72
**ANGIOGRAPHIC VARIABLES**				
TICI ≥ 2B	9.39 (1.92–45.80)	0.005	
Stenosis acutely treated	1.68 (0.39–7.07)	0.47	6.30 (1.11–35.67)	0.03
Distal access catheter	2.29 (0.58–8.94)	0.23	16.25 (3.06–86.41)	0.001
Atheromatous lesion	0.78 (0.18–3.41)	0.74	1.27 (0.29–5.53)	0.74
**OCCLUSION SITE**				
Carotid-T	0.41 (0.10–1.64)	0.2	0.21 (0.05–0.90)	0.003
**POSTPROCEDURAL CHARACTERISTICS**				
PH2 n/N	N/A^*^		
Delayed treatment of ICA	4.54 (0.45–45.86)	0.19		

Significant values are indicated with bold font.

* *Small patient numbers with infinite confidence intervals*.

Table 4 shows the analysis of factors in patients with successful and unsuccessful recanalization. Of note, distal access catheters were used more often in patients with successful recanalization (78.9 vs. 18.8%; *p* = 0.0006) and in those in whom the cervical lesion was treated more often in the acute phase by PTA with or without stenting (47.4 vs. 18.8%; *p* = 0.035). Thrombus localization at the carotid-T was observed more frequently in patients with unsuccessful recanalization (62.5 vs. 26.3%; *p* = 0.0442). The etiology of the cervical lesion (dissecting vs. atheromatous) was not significantly different between patients with successful and unsuccessful recanalization (*p* = 1.0). Univariate regression analysis confirmed these results as the only factors significantly associated with successful revascularization with acute treatment of the stenosis by stenting and/or PTA (OR 6.3, 95% CI 11–35.67, *p* = 0.03) and, even more importantly, the use of distal access catheters (OR 16.25, 95% CI 3.06–86.41, *p* = 0.001). Occlusion of the carotid-T hindered successful recanalization compared to occlusion of the MCA (OR 0.21, 95% CI 0.05–0.90, *p* = 0.003).

**Table 4:** Successful vs. unsuccessful recanalization.

	**Unsuccessful recanalization**	**Successful recanalization**	***P*–value**
**PATIENT CHARACTERISTICS**			
Male sex n (%)	13/16 (81.3%)	17/19 (89.5%)	0.64
Age (mean ± SD)	65.9 ± 12.8	63.5 ± 9.8	0.53
Baseline NIHSS (mean ± SD)	17.8 ± 3.5	16.9 ± 5.2	0.6
Onset-to-groin time (mean ± SD)	264.1 ± 41.1	248.2 ± 61.9	0.39
Intravenous thrombolysis n (%)	11/16 (68.8%)	12/19 (63.2%)	1.0
**ANGIOGRAPHIC VARIABLES**			
Stenosis acutely treated n/N (%)	2/16 (12.5%)	9/19 (47.4%)	0.035
Distal access catheter n/N (%)	3/16 (18.8%)	15/19 (78.9%)	<0.001
Atheromatous lesion n/N (%)	11/16 (68.8%)	14/19 (73.7%)	1.0
**OCCLUSION SITE**			
Carotid-T n/N (%)	10/16 (62.5%)	5/19 (26.3%)	0.044

*Significant differences are indicated with bold font*.

## Discussion

While the indication for MT of acute tandem occlusions of the anterior circulation is generally undisputed (4), it remains unclear how the cervical lesion should be treated. The vast majority of publications to date focus on stenting of the cervical lesion, with little data on the results of PTA-only and conservative approaches during the acute phase (and, where indicated, delayed treatment of the lesion), as noted by Wilson et al. (12) in their recent meta-analysis.

Our single center series suggests that a conservative approach for the ICA lesion is feasible and that the rate of clinically favorable outcome (45.7%) is within the range of previously published large-scale data on MT (18). Of note, neither group comparisons nor logistic regression analyses indicated that the treatment approach for cervical lesion during MT affected the patient's clinical outcome. Favorable outcome was predicted by a higher preinterventional ASPECT score and even more importantly by successful recanalization of the intracranial occlusion, which was achieved in 54.3% of the included patients. Successful recanalization was strongly favored by the use of distal access catheters, which, when these devices were employed, resulted in successful recanalization in 78.9% of patients, However, it should be noted that successful recanalization was also significantly more frequent when the carotid lesion was treated by stenting and/or balloon PTA during the acute phase. In a recent paper by Papanagiotou et al. (14) on the Thrombectomy In Tandem Lesions (TITAN) study collective, a multicentric registry on patients with MT for tandem stroke, successful recanalization was observed more frequently in patients with acute treatment of the ICA lesion (stent and/or PTA during MT) than in those with conservative management (79.4 vs. 60%) (14). The authors also found that patients without acute treatment of the cervical lesion were less likely to experience a favorable outcome after 3 months (42 vs. 58%). The results of another study on the same patient population were similar to ours, as extracranial carotid stenting had no significant impact on patient outcome which was, among other factors, predicted by age, NIHSS and baseline ASPECTS (19). However, that study confirmed a link between acute carotid stenting and successful reperfusion. Nevertheless, the assumption that stenting is definitively associated with successful reperfusion and that “the ICA lesion should be always treated” (14) during MT should be viewed with caution as both our single-center series and the TITAN study are entirely retrospective data with self-reported clinical outcome measures. One should bear in mind that neurointerventionists are probably more aggressive in treating large vessel occlusions—both intra- and extra-cranially—when a favorable outcome is anticipated so that these results are likely subject to selection bias (20). Moreover, there are multiple personal preferences in the performance of MT that may vary between institution and physicians; additionally, a physician's preference likely continually adapts as a result of personal experience. The way we interpret our data is that a conservative approach for the ICA lesion during MT with delayed treatment offers favorable outcomes for a significant proportion of patients. Successful recanalization is, however, crucial for clinical success and if the ICA lesion cannot be passed with a distal access catheter, the interventionist's threshold for performing a PTA with our without concomitant stenting should be low. Even with a conservative protocol, PTA or stenting of the cervical lesion was still considered necessary in 22.9 and 8.6% of patients, respectively. Furthermore, the fact that acute treatment of the proximal ICA lesion was carried out in 11 patients precludes definitive statements on this treatment strategy.

Unfortunately, most studies do not provide data on the delayed management of the ICA lesion if it was not stented during MT. Our data indicate that a conservative approach for the ICA lesion during the acute phase may reduce the number of overtreatments. Treatment outside the acute phase by endarterectomy or stenting was indicated in only 35% of surviving patients with an atheromatous ICA lesion. In another publication on carotid stenting after MT, the rate of delayed ICA stenting was 66.4%, which led the authors to conclude that this approach may help to prevent futile treatments (10). However, the hemorrhagic risks of stenting during MT must be balanced with the risk of leaving a “non-protected,” potentially emboligenic cervical lesion behind. This risk may be particularly high in patients with a free floating thrombus (21). In our study, no recurrent ischemic events were observed between the acute phase and the definitive treatment of the carotid stenosis after a mean of 41.7 days. Another study postulated that the risk of recurrent ipsilateral ischemic stroke in patients with symptomatic carotid artery stenosis awaiting endarterectomy is as high as 11.5% at 14 days and 18.8% at 90 days (22). However, disabling or fatal strokes at 90 days occurred in only 16 of 377 patients in that analysis (4.2%). A possible explanation for the differences between the latter series and our data may be the rather small sample size of our study. One could also hypothesize that in patients after MT, in whom—depending on the inclusion criteria—considerable infarcts may be present, recurrent emboli may stay silent if they find their way in the already infarcted territory. Another explanation is that we also included 10 patients with carotid artery dissections in whom the risk of recurrent embolism is known to be low (23). Furthermore, even if no active treatment of the ICA lesion by PTA or stenting is performed, passing the lesion with large-bore aspiration or guide catheters may result in a partial dilatation of the stenosis, akin to the Dotter technique (24).

Parenchymal hematoma occurred in 11.4% of our patients (5.7% PH1 and PH2 each), which is within the range of larger series on carotid stenting during MT. For example, Gory et al. (19) observed rates of 8.7% for PH1 and 5.1% for PH2 in the TITAN collective (66.2% received a carotid stent during MT), whereas Behme et al. (5) reported rates of 8 and 9% of PH1 and PH2 in a study in which all patients underwent carotid stenting during MT. Papanagiotou et al. (14) did not report on diminished hematoma occurrence in non-stented patients. Whether the avoidance of stenting effectively diminishes the risk of intracranial hemorrhage thus remains to be proven.

No effect of the proximal lesion's etiology (dissection vs. atheroma) on outcome or recanalization was observed in the present study, which is in line with a previous publication on the TITAN patient collective data (25).

We observed a trend toward a favorable outcome when IV thrombolysis was used, which may support the results of another recent publication (19). However, interpretation of this finding is likely limited by significant selection bias. Moreover, with the Solitaire With the Intention For Thrombectomy Plus Intravenous t-PA Versus DIRECT Solitaire Stent-retriever Thrombectomy in Acute Anterior Circulation Stroke (SWIFT DIRECT) trial actively recruiting (www.ClinicalTrials.gov NCT03192332), a randomized controlled trial will hopefully help to define the current role of IV thrombolysis in stroke treatment.

Our study has limitations, the retrospective design and rather small sample size being most important, which limits the generalizability of the findings. The combined report on atheromatous lesions and carotid artery dissection may contribute to a less homogeneous patient sample. However, the two entities are commonly reported together in the literature (14) and both our results and another study confirm that angiographic and clinical outcomes after MT do not differ between both lesion types (25). Furthermore, the rate of complete recanalization obtained at our center appears low compared to the recent literature; however, this can be explained by a very strict independent evaluation and the use of the stricter TICI scale instead of the modified TICI scale. Importantly, an evolution in practitioners' experience and in technology is highly likely as the first patients in our database were treated in 2010. Unfortunately, we did not have clinical data on the symptomatology of post-interventional intracranial hemorrhage available to us. Nonetheless, it was later shown that PH2 is a reliable, entirely imaging-based predictor of unfavorable outcome (26).

## Conclusion

Our study shows that a conservative approach for the ICA lesion during MT is feasible and offers favorable outcomes for a significant number of patients. Factors influencing patient outcome are the size of the baseline ischemic core and particularly the result of recanalization. The utilization of distal access catheters seems highly beneficial for successful recanalization; however, if the cervical lesion cannot be passed with such a device, the threshold for PTA with our without concomitant stenting should be low as it may facilitate intracranial revascularization. Acute treatment of the proximal ICA lesion had no positive effect on clinical outcome, which may be explained by our sample size and probable selection bias. Even if a conservative treatment is planned, acute PTA or stenting of the cervical lesion in order to gain access to the intracranial vasculature may be necessary in a considerable proportion of patients. Beyond that, avoiding treatment of the proximal ICA lesion during the acute phase may help to reduce overtreatments. Altogether, our study provides valuable information on a much-debated pathology and highlights the lack of clarity, which will only be resolved with a properly designed randomized controlled trial.

## Author contributions

All authors listed have made a substantial, direct and intellectual contribution to the work, and approved it for publication.

### Conflict of interest statement

LP consults for Balt, Cerenovus, Microvention, Penumbra and Vesalio. The remaining authors declare that the research was conducted in the absence of any commercial or financial relationships that could be construed as a potential conflict of interest.
